# Salivary Oxidative-Antioxidant Profile Following Adjunctive Gaseous Ozone Administration in Non-Surgical Periodontal Treatment: A Randomized Controlled Trial

**DOI:** 10.3390/jcm13175272

**Published:** 2024-09-05

**Authors:** Biagio Rapone, Antonio Scarano, Erda Qorri, Alessia Pardo, Giovanna Murmura, Alessandro D’Albenzio, Elisabetta Ferrara

**Affiliations:** 1 Interdisciplinary Department of Medicine, University of Bari “Aldo Moro”, 70121 Bari, Italy; 2 Department of Medical, Oral and Biotechnological Sciences, University “G. d’Annunzio” of Chieti-Pescara, 66100 Chieti, Italy; ascarano@unich.it; 3 Department of Dentistry, Faculty of Medical Sciences, Albanian University, 1001 Tirana, Albania; e.quorri@albaninanuniversity.edu.al; 4 Dentistry and Maxillofacial Surgery Unit, Department of Surgery, Dentistry, Pediatrics and Gynecology (DIPSCOMI), University of Verona, 37134 Verona, Italy; alessia.pardo@univr.it; 5 Department of Innovative Technologies in Medicine and Dentistry, University “G. d’Annunzio” of Chieti-Pescara, 66100 Chieti, Italy; giovanna.murmura@unich.it; 6 Complex Operative Unit of Pathological Addiction, Addiction Service, ASL2 Abruzzo, 66100 Chieti, Italy; alessandro.dalbenzio@asl2abruzzo.it; 7 Department of Human Sciences, Law, and Economics, Telematic University “Leonardo da Vinci”, UNIDAV, Torrevecchia Teatina, 66100 Chieti, Italy

**Keywords:** ozone administration, periodontitis, non-surgical periodontal treatment, oxidative stress, malondialdehyde, total antioxidant capacity, superoxide dismutase

## Abstract

**Background**: Periodontitis is associated with increased oxidative stress, which may impair treatment outcomes. Ozone therapy has shown promise in reducing oxidative stress and improving periodontal health. This study examined the impact of adjunctive gaseous ozone administration on salivary oxidative stress markers in patients with periodontitis stages II–IV and grades A–C undergoing non-surgical periodontal treatment (NSPT). **Methods**: Ninety patients with periodontitis were randomly allocated to either the test group (NSPT with gaseous ozone administration) or the control group (NSPT alone) using computer-generated randomization. The OzoneDTA system was used to deliver ozone at 2100 ppm for 60 s per site once weekly for 4 weeks. Clinical periodontal parameters (probing depth [PD], clinical attachment level [CAL], plaque index [PI], gingival index [GI]) and salivary oxidative stress markers (malondialdehyde [MDA], total antioxidant capacity [TAC], superoxide dismutase [SOD]) were assessed by blinded examiners at baseline, 3, and 6 months post-treatment. **Results**: Mixed ANOVA revealed significant three-way interactions between time, treatment, and stage or grade for clinical and biochemical measures (*p* < 0.001). The test group exhibited significant improvements in TAC (mean difference: 0.45 ± 0.12 mmol/L, *p* = 0.002), MDA (−0.38 ± 0.09 nmol/mL, *p* = 0.001), and SOD (65 ± 18 U/mL, *p* < 0.001) compared with the control group, with more pronounced effects in stages III and IV. Large effect sizes (Cohen’s d > 0.8) were observed for the test group between baseline and 6 months for all markers. **Conclusions**: Gaseous ozone administration as an adjunct to NSPT can effectively improve clinical periodontal parameters and salivary oxidative stress markers, particularly in stages III and IV periodontitis. The enhanced outcomes may be attributed to ozone’s antimicrobial and immunomodulatory properties, which synergistically reduce oxidative stress and promote periodontal healing.

## 1. Introduction

Periodontitis, a chronic inflammatory disease affecting the supporting structures of teeth, represents a significant global health concern. With a prevalence ranging from 20% to 50% in adults worldwide and severe forms impacting approximately 11% of the population, its ramifications extend beyond oral health [1]. The condition has been associated with various systemic ailments, including cardiovascular diseases, diabetes, and adverse pregnancy outcomes, underscoring its importance as a public health issue [2,3]. The pathogenesis of periodontitis involves a complex interplay between the microbial biofilm and the host immune response. Central to this process is oxidative stress, which plays a crucial role in tissue destruction and disease progression. Oxidative stress occurs when there is an imbalance between the production of reactive oxygen species (ROS) and the body’s ability to neutralize them through antioxidant mechanisms. ROS, including superoxide (O_2_•−), hydrogen peroxide (H_2_O_2_), and hydroxyl radicals (OH•), are highly reactive molecules that can damage cellular components such as lipids, proteins, and DNA [4,5].

In healthy periodontal tissues, a delicate balance exists between ROS production and antioxidant defense mechanisms. However, in periodontitis, this equilibrium is disrupted. The inflammatory response to periodontal pathogens leads to an increased production of ROS by activated neutrophils and macrophages [6,7]. Simultaneously, the antioxidant capacity of periodontal tissues may be overwhelmed, resulting in a state of oxidative stress.

The consequences of oxidative stress in periodontal tissues are far-reaching and multifaceted. Reactive oxygen species (ROS) initiate a cascade of damaging events throughout the cellular structures. They attack polyunsaturated fatty acids in cell membranes, triggering lipid peroxidation and producing byproducts like malondialdehyde (MDA), which compromise membrane integrity and impair cellular function [1,8,9,10]. Simultaneously, ROS modify amino acid residues in proteins, causing fragmentation or cross-linking that disrupts enzyme activity and cellular signaling pathways. The assault extends to the genetic level, where oxidative stress induces DNA strand breaks and base modifications, potentially leading to mutations and cellular dysfunction [11,12,13]. Furthermore, ROS activate pro-inflammatory transcription factors such as nuclear factor-κB (NF-κB), setting in motion a self-perpetuating cycle of inflammation through increased cytokine expression [14]. This intricate web of oxidative damage underlies the progressive nature of periodontal disease, contributing to tissue destruction and impaired healing responses [7,15,16,17]. To counteract the harmful effects of ROS, the body employs a complex network of antioxidant defense mechanisms. These include enzymatic antioxidants such as superoxide dismutase (SOD), catalase, and glutathione peroxidase, as well as non-enzymatic antioxidants like glutathione, vitamin C, vitamin E, and carotenoids [18]. The total antioxidant capacity (TAC) of biological fluids, including saliva, represents the cumulative effect of all antioxidants present.

Non-surgical periodontal treatment (NSPT), primarily consisting of scaling and root planing (SRP), remains the cornerstone of periodontal therapy [1,2,3]. SRP aims to remove the bacterial biofilm, calculus, and contaminated cementum from the root surface, thereby reducing the microbial load and promoting healing [18]. However, NSPT has several limitations, including incomplete removal of subgingival biofilm, especially in deep pockets and furcation areas; the potential for rapid recolonization of treated sites by periodontal pathogens; limited efficacy in modulating the host immune response and oxidative stress; and reduced effectiveness in advanced cases or in patients with compromised healing responses [19].

These limitations have led to the exploration of adjunctive therapies to enhance treatment outcomes. Ozone therapy has emerged as a promising adjunctive treatment in periodontics. Ozone (O_3_) is a triatomic molecule composed of three oxygen atoms, characterized by its high oxidative potential and unique biological properties.

The mechanisms of action of ozone in periodontal therapy are multifaceted.

Antimicrobial effect: Ozone exhibits potent antimicrobial activity against a wide range of periodontal pathogens by oxidizing bacterial cell membranes, leading to cell lysis, disrupting viral capsids, and inhibiting fungal growth [20].

Immunomodulation: Ozone modulates the immune response by stimulating the production of immunocompetent cells; enhancing the release of cytokines such as interferon-γ, tumor necrosis factor-α, and interleukin-2; and activating macrophages and neutrophils.

Oxidative stress regulation: Paradoxically, ozone can reduce oxidative stress by activating the nuclear factor erythroid 2-related factor 2 (Nrf2) pathway, which upregulates antioxidant enzymes, and by inducing mild oxidative stress, triggering an adaptive response that enhances overall antioxidant capacity [21].

Improved tissue oxygenation: Ozone therapy can enhance tissue oxygenation by increasing the oxygen-carrying capacity of blood and stimulating the production of adenosine triphosphate (ATP) in cells [20,21].

Enhanced wound healing: Ozone promotes wound healing by stimulating the proliferation of fibroblasts and epithelial cells and increasing the production of growth factors [21].

In dentistry, ozone can be applied using various delivery systems and forms. Gaseous ozone is delivered through specialized handpieces with silicone cups to isolate the treatment area. Ozonated water is used for irrigation of periodontal pockets or as a mouthwash. Ozonated oils are applied topically as a gel or ointment. Each form has its advantages and specific applications in periodontal therapy [22].

Despite the potential benefits of ozone therapy, its efficacy in different stages and grades of periodontitis, as defined by the 2017 World Workshop on the Classification of Periodontal and Peri-Implant Diseases and Conditions, remains unclear [23]. This knowledge gap is critical, as the new classification system provides a framework for personalized treatment approaches based on disease severity and progression risk.

The aim of this study was to evaluate the efficacy of gaseous ozone therapy as an adjunct to NSPT in patients with periodontitis stages II–IV and grades A–C. We hypothesized that adjunctive ozone therapy would provide greater improvements in both clinical periodontal parameters and salivary oxidative stress markers compared with NSPT alone, with more pronounced effects in the advanced stages and higher grades of periodontitis.

Specifically, we aimed to assess clinical periodontal parameters including probing depth (PD), clinical attachment level (CAL), plaque index (PI), and gingival index (GI). Additionally, we evaluated salivary oxidative stress markers, namely, malondialdehyde (MDA) as a marker of lipid peroxidation, total antioxidant capacity (TAC), and superoxide dismutase (SOD) activity.

By evaluating both the clinical and biochemical outcomes, we aimed to provide a more nuanced understanding of ozone therapy’s potential in periodontal treatment. This approach aligns with the growing emphasis on personalized medicine in dentistry, potentially informing tailored treatment strategies based on individual patient characteristics and disease presentation.

## 2. Materials and Methods

### 2.1. Study Design and Ethics

This study was designed as a randomized, controlled, parallel-group clinical trial conducted at the Clinic of Periodontology of the Faculty of Dental Medicine of Albanian University. The study protocol was approved by the Institutional Review Board at Albanian University (reference number 2018/224) and was conducted in accordance with the Good Clinical Practice guidelines and the Declaration of Helsinki (1975), as revised in 2013. The study was registered in a clinical trial registry (Registration No. ISRCTN17281691), and the study design followed the CONSORT guidelines. Participants were recruited from the patient pool at the University Dental School.

**Figure 1:**
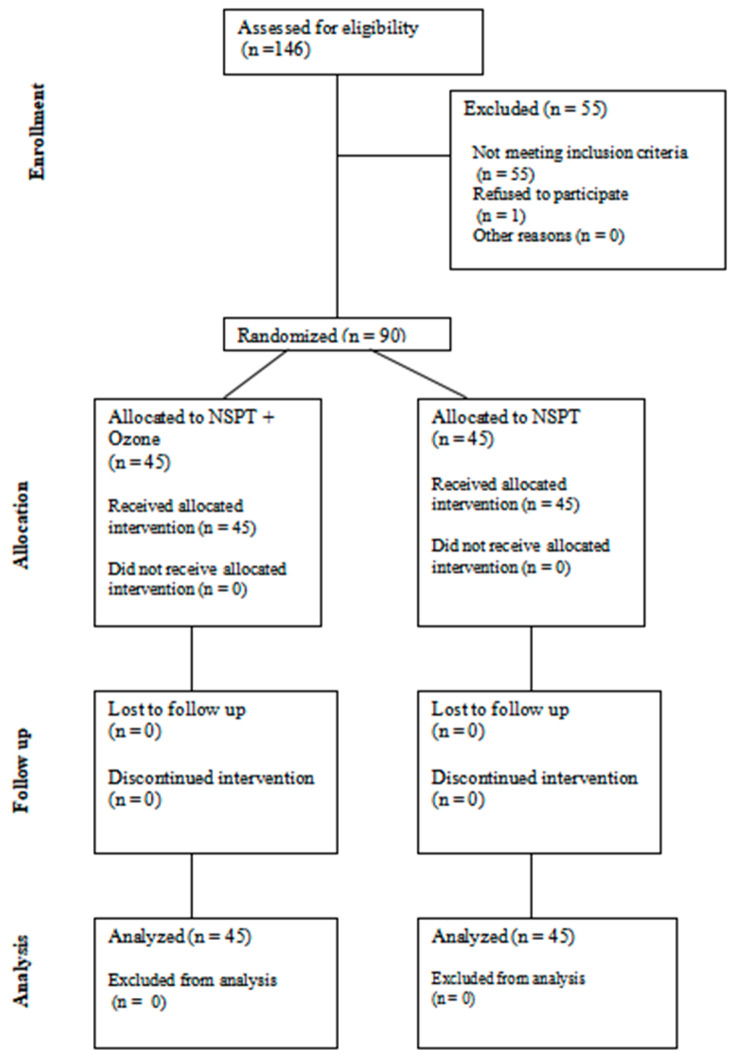
CONSORT flow diagram of participant selection and randomization.

All eligible participants were provided with detailed information about the study, including its purpose, procedures, potential risks, and benefits. Written informed consent was obtained from all participants prior to their enrollment in the study. Participants were informed of their right to withdraw from the study at any time without any consequences to their future treatment. Figure 1 illustrates the CONSORT flow diagram detailing the process of participant selection, randomization, and progression through the various stages of the study.

### 2.2. Participants

Ninety systemically healthy patients with periodontitis, aged 35–60 years, were recruited for this study. Inclusion criteria were as follows:

1. Diagnosis of periodontitis stages II, III, or IV, with the presence of at least 20 teeth;
2. Interdental clinical attachment loss (CAL) detectable at ≥ 2 non-adjacent teeth or buccal or oral CAL ≥ 3 mm with pocketing ≥ 3 mm detectable at ≥ 2 teeth;
3. No periodontal treatment in the past 6 months.

Exclusion criteria included systemic diseases, antibiotic use in the past 6 months, pregnancy or lactation, and presence of acute periodontal lesions.

Eligibility criteria are detailed in Table 1.

Participants were stratified based on the stage (II–IV) and grade (A–C) of periodontitis according to the 2017 World Workshop on the Classification of Periodontal and Peri-Implant Diseases and Conditions [23]. Staging was determined by the severity and complexity of the disease, while grading was based on the risk of progression and potential for treatment response (Table 2).

Our study included patients with periodontitis stages II–IV and grades A–C to evaluate ozone therapy efficacy across various disease severities and progression rates. This stratification allowed us to analyze treatment effects based on the initial disease presentation. The participant selection process followed the guidelines outlined in the CONSORT (Consolidated Standards of Reporting Trials) flow diagram. At the end of the screening process, a total of 91 participants were initially enrolled in the study. Of these, 1 participant withdrew consent during the initial enrollment phase, leading to a final total of 90 participants who completed the study.

### 2.3. Outcome Measure Assessment

To comprehensively evaluate the efficacy of the interventions, we employed a multifaceted approach to outcome measurement. This approach encompassed clinical periodontal parameters, biochemical markers of oxidative stress, and patient-reported outcomes. The selection of these measures was based on their established relevance in periodontal research and their potential to provide insights into both the clinical and biological effects of the treatments.

#### 2.3.1. Primary Outcomes

A comprehensive full-mouth examination (FME) was conducted for all participants to evaluate the following primary and secondary parameters.

1. Clinical Periodontal Parameters

(a) Probing depth (PD), in mm;
(b) Clinical attachment level (CAL), in mm;
(c) Plaque index (PI), assessed on a scale of 0–3 (Silness and Löe 1964 [5]);
(d) Gingival index (GI), evaluated on a scale of 0–3 (Löe and Silness 1963 [4]).

2. Salivary Oxidative Stress Markers

(a) Malondialdehyde (MDA), quantified in nanomoles per milliliter (nmol/mL);
(b) Total antioxidant capacity (TAC), measured in millimoles per liter (mmol/L);
(c) Superoxide dismutase (SOD) activity, expressed in units per milliliter (U/mL).

#### 2.3.2. Secondary Outcome Measures

1. Clinical Parameters

(a) Bleeding on probing (BOP), calculated as a percentage of sites;
(b) Tooth mobility, assessed using Miller’s classification (0–3).

All clinical parameters were measured at six sites per tooth (mesiobuccal, midbuccal, distobuccal, mesiolingual, midlingual, and distolingual) using a UNC-15 periodontal probe (Hu-Friedy, Chicago, IL, USA). Measurements were recorded to the nearest millimeter.

Salivary samples for oxidative stress marker analysis were collected at baseline, 3 months, and 6 months post-treatment. The specific assays and analytical methods for each marker are detailed in the biochemical analysis section.

### 2.4. Examiners’ Calibration and Agreement

Prior to the study, two examiners (E.Q., B.R.) underwent a calibration process on 10 patients not included in the study sample. The calibration was performed in two sessions, one week apart. Intra- and inter-examiner agreement was evaluated using the intraclass correlation coefficient (ICC) for PD and CAL and Cohen’s kappa coefficient (κ) for PI and GI.

#### Calibration Process

Session 1: Both examiners independently measured PD, CAL, PI, and GI on all teeth of 5 patients.

Session 2: One week later, the measurements were repeated on the same 5 patients, plus 5 new patients.

Session 3: In the third week, measurements were taken on 5 new patients.

For each session, the examiners were blinded to each other’s measurements. After each session, results were compared, and any discrepancies were discussed to reach a consensus on measurement techniques. Intra-examiner reliability was assessed by comparing each examiner’s measurements from sessions 1 and 2 for the initial 5 patients. Inter-examiner reliability was evaluated by comparing the measurements between examiners for all 10 patients across the three sessions. The intraclass correlation coefficient (ICC) was used to assess agreement for continuous variables (PD and CAL), while Cohen’s kappa (κ) was used for categorical variables (PI and GI). The calibration was considered successful when ICC values exceeded 0.8 and κ values exceeded 0.6. Table 3 displays the intra- and inter-examiner agreement values.

The high ICC and κ values demonstrated excellent intra- and inter-examiner reliability, ensuring the consistency and accuracy of the clinical measurements throughout the study.

### 2.5. Randomization and Blinding

Participants were randomly assigned in a 1:1 ratio to either the test group (NSPT + gaseous ozone therapy) or the control group (NSPT alone). The randomization sequence was generated using a computer-based random number generator (Random.org) by an independent statistician not involved in the study. Block randomization with a block size of 6 was used to ensure balanced allocation throughout the study period. The allocation sequence was implemented using sequentially numbered, opaque, sealed envelopes (SNOSEs). Each envelope contained a card indicating the assigned treatment group. The envelopes were prepared by an independent research assistant who was not involved in any other aspect of the study. This process ensured that the allocation sequence was concealed from the researchers enrolling participants. Due to the nature of the interventions, complete blinding of participants was not possible. However, participants were not informed about the specific hypothesis being tested, to minimize potential bias. The study examiners (E.Q. and B.R.) performing the clinical assessments were blinded to the treatment allocation. They were not involved in the treatment process and were instructed not to ask participants about their treatment. The statistician analyzing the data was blinded to the group assignments. All data were coded before analysis, with group identities revealed only after the completion of the analysis.

The clinicians administering the NSPT and ozone therapy could not be blinded due to the nature of the interventions. However, they were not involved in the clinical assessments or data analysis to minimize potential bias. To maintain blinding, the following measures were implemented: all participants were instructed not to disclose their treatment to the clinical examiners; treatment sessions were scheduled separately from assessment sessions; all study documents used participant identification numbers rather than group assignments; and the randomization code was kept in a secure location and was broken only after the completion of data analysis.

### 2.6. Treatment Protocol

#### 2.6.1. Non-Surgical Periodontal Treatment (NSPT)

All participants received full-mouth scaling and root planing (SRP) under local anesthesia. The treatment was performed by experienced periodontists using both hand instruments (Gracey curettes, Hu-Friedy, Chicago, IL, USA) and ultrasonic devices (Piezon Master 700, EMS, Nyon, Switzerland).

SRP Sessions:

Frequency: 2 to 4 sessions, based on disease severity and extent

Duration: 60–90 min per session

Interval: 7 days between sessions

Treatment period: Completed within 28 days

#### 2.6.2. Gaseous Ozone Therapy (Test Group Only)

Participants in the test group received adjunctive ozone therapy using the OzoneDTA system (Sweden and Martina, Padua, Italy).

Ozone Therapy Parameters:

Concentration: 2100 ppm

Flow rate: 615 cc/min

Application duration: 60 s per site

Delivery system: Handpiece with silicone cup (Probe no. 1, 10° angle, OZONE203011; Probe no. 2, 50° angle, OZONE203012)

Treatment Schedule:

Initial application immediately following each SRP session

Follow-up applications at 7, 14, and 28 days post-SRP

Ozone Therapy Protocol:

Phase 1 (Initial Treatment):

Two-minute oral rinse with ozonated water (1:3 dilution)

Full-mouth decontamination and topical irrigation with ozonated water

Application of 1–2 cycles of ozone gas (intensity 8–10) to pathological pockets under local anesthesia

Phase 2 (During SRP):

Quadrant-wise root planing

Two-minute rinse with ozonated water (1:3 dilution) after each quadrant

Deplaquing

Application of 1–2 cycles of ozone gas (intensity 8–10) to treated quadrant’s pathological pockets under local anesthesia

Phase 3 (Maintenance):

Initiated 14 days post SRP completion

Two-minute ozonated water rinse (1:3 dilution)

Deplaquing

Application of 1–2 cycles of ozone gas (lower intensity 4–5) to all pathological pockets

Table 4 presents the treatment schedule for both groups, illustrating the timing of interventions and assessments throughout the study period.

### 2.7. Salivary Sample Collection and Biochemical Analysis

**Figure 2:**
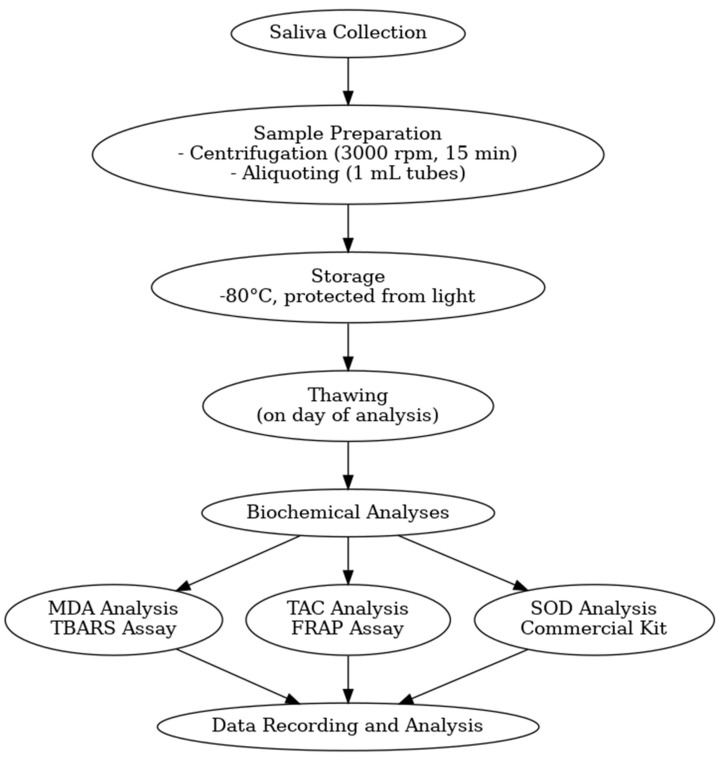
Flowchart of saliva collection and analysis process.

Unstimulated whole saliva samples were collected from participants at baseline, 3 months, and 6 months post-treatment following written informed consent and Institutional Ethics Committee approval. The collection protocol adhered to standardized procedures to ensure sample integrity and consistency. Figure 2 outlines the key steps in the saliva collection and analysis process, from initial collection through to the specific assays used for each biomarker.

Participants were instructed to refrain from eating, drinking, and performing oral hygiene for a minimum of 2 h prior to sample collection. Non-stimulated saliva was collected in the morning using the spitting method, which minimizes potential confounding factors associated with stimulated saliva collection techniques.

#### 2.7.1. Sample Processing and Storage

Saliva samples were collected in ice-chilled vials (−20 °C) to preserve biochemical integrity. Samples were then centrifuged at 3000 rpm for 15 min to remove cellular debris and particulate matter. The supernatant was carefully aliquoted into 1 mL microcentrifuge tubes and stored at −80 °C, protected from light, to prevent degradation of biomarkers until the time of analysis.

#### 2.7.2. Biochemical Analysis

Three key biomarkers were analyzed to assess oxidative stress and antioxidant capacity, as explained in Table 5.

The measurement of TAC and SOD activity in saliva provides valuable insights into the redox status of oral tissues. Unlike some biomarkers that reflect single oxidative events, these parameters offer a more comprehensive view of the oxidative stress–antioxidant balance. SOD, along with catalase and glutathione peroxidase, constitutes the primary enzymatic antioxidant defense in human saliva and serum [24]. SOD specifically scavenges superoxide radicals, converting them to hydrogen peroxide. This action is crucial in mitigating oxidative damage, as evidenced by studies showing that human SOD overexpression decreases lipid peroxidation. Conversely, mutations in SOD have been associated with an increased risk of age-related diseases, including neurodegenerative disorders and cardiovascular diseases. TAC measurement serves as a suitable screening tool for overall antioxidant status. It has been correlated with various pathological conditions and provides a comprehensive assessment of antioxidant capacity.

### 2.8. Assessment of Adverse Events

The occurrence of adverse events was systematically monitored throughout the study period. Adverse events were defined as any unfavorable and unintended sign, symptom, or disease temporally associated with the use of the study intervention, whether considered related to the intervention or not.

Participants were instructed to report any unusual symptoms or discomfort experienced during or after the treatment sessions. At each follow-up visit (baseline, 3 months, and 6 months), patients were specifically asked about any adverse experiences using a standardized questionnaire. The questionnaire included both open-ended questions and a checklist of potential side effects associated with periodontal treatment and ozone therapy.

For each reported adverse event, the following information was recorded:

Nature of the event;

Onset and duration;

Severity (mild, moderate, or severe);

Relationship to the study intervention (not related, possibly related, probably related, or definitely related);

Action taken (none, temporary discontinuation of treatment, permanent discontinuation of treatment);

Outcome (resolved, ongoing, sequelae).

All adverse events were reviewed by the study dentist to assess their potential relationship with the study intervention. Serious adverse events, defined as any untoward medical occurrence that results in death, is life-threatening, requires hospitalization or prolongation of existing hospitalization, results in persistent or significant disability/incapacity, or is a congenital anomaly/birth defect, were to be reported to the Institutional Review Board within 24 h of awareness.

The frequency and nature of adverse events were compared between the test and control groups to evaluate the safety profile of the adjunctive ozone therapy.

### 2.9. Statistical Analysis

#### 2.9.1. Sample Size Calculation

The sample size calculation was based on the following primary outcome variables (Table 6): salivary malondialdehyde (MDA), total antioxidant capacity (TAC), and superoxide dismutase (SOD). We used the following formula for two-group comparisons of continuous outcome [26]:

n = 2 × (Zα/2 + Zβ)^2^ × σ^2^/Δ^2^

where n = sample size per group, Zα/2 = 1.96 (for α = 0.05, two-tailed), Zβ = 0.84 (for 80% power), σ = standard deviation of the outcome variable, and Δ = expected mean difference between groups.

Assumptions for each outcome variable were based on previous studies investigating the effect of periodontal treatment on salivary oxidative stress markers [17].

The largest calculated sample size (36 per group, rounded up from 35.28) was selected to ensure adequate power for all primary outcomes. To account for potential dropouts, we adjusted the sample size assuming a 20% dropout rate:

Adjusted n = n/(1 − dropout rate) = 36/(1 − 0.2) ≈ 45 per group

To further ensure an adequate sample size and balance the number of participants across the stage and grade subgroups, we recruited 45 participants per group, resulting in a total sample size of 90 participants.

#### 2.9.2. Data Analysis

Statistical analysis was performed using SPSS software (version 25.0; IBM Corp., Armonk, NY, USA). The Shapiro–Wilk test was used to assess the normality of data distribution. Descriptive statistics were presented as mean ± standard deviation (SD) for normally distributed continuous variables, median (interquartile range) for non-normally distributed continuous variables, and frequencies (percentages) for categorical variables.

For the primary outcome variables (MDA, TAC, and SOD levels), between-group comparisons were conducted using independent samples t-tests or Mann–Whitney U tests, depending on data distribution. Changes within each group over time (baseline, 3 months, and 6 months) were evaluated using repeated measures ANOVA or Friedman’s test, as appropriate. Post-hoc pairwise comparisons were performed using Bonferroni correction or Wilcoxon signed-rank tests.

Secondary outcome variables (clinical periodontal parameters: PD, CAL, PI, and GI) were analyzed using the same statistical approaches as the primary outcomes.

Subgroup analyses were conducted to assess the efficacy of interventions based on periodontitis stage (II–IV) and grade (A–C). Within each subgroup, oxidative stress parameters and clinical periodontal parameters were compared between test and control groups using appropriate statistical tests.

The association between oxidative stress parameters and clinical periodontal parameters was evaluated using Pearson’s or Spearman’s correlation coefficients, depending on data distribution.

Multiple linear regression analysis was performed to identify factors influencing changes in oxidative stress parameters, considering potential confounders such as age, gender, and baseline clinical parameters.

To adjust for multiple comparisons, the Bonferroni correction was applied to the significance level for primary outcome variables. With three primary outcomes, the adjusted significance level was set at 0.05/3 = 0.0167. This correction helps control the familywise error rate and reduces the risk of type I errors when testing multiple hypotheses simultaneously.

All statistical tests were two-tailed. A *p*-value < 0.0167 was considered statistically significant for the primary outcome variables, while a *p*-value < 0.05 was considered significant for the secondary outcome variables and subgroup analyses. The 95% confidence intervals (CIs) were calculated for the mean differences in oxidative stress parameters and clinical periodontal parameters between the groups.

#### 2.9.3. Subgroup Analysis

Participants were stratified into subgroups according to their stage (II–IV) and grade (A–C) of periodontitis. Within each subgroup, the oxidative stress parameters and clinical periodontal parameters were compared between the test and control groups using the same statistical tests as described for the primary and secondary outcomes. This approach allowed us to assess whether the efficacy of the interventions varied based on the severity and progression rate of periodontitis. Subgroup analyses were performed using two-way mixed ANOVAs with the treatment group as a between-subjects factor and time as a within-subjects factor. Separate analyses were conducted for each outcome variable (PD, CAL, PI, GI, MDA, TAC, and SOD) within the following subgroups: age groups (<45 years, 45–55 years, > 55 years), gender (male, female), smoking status (never smokers, former smokers), periodontitis stage (II, III, IV), and periodontitis grade (A, B, C).

#### 2.9.4. Multiple Linear Regression Analysis

A multiple linear regression analysis was performed to identify factors influencing the changes in oxidative stress parameters. The dependent variables were the changes in MDA, TAC, and SOD levels from baseline to 6 months. Independent variables included treatment group (test vs. control), age, gender, smoking status, baseline clinical parameters (PD, CAL, PI, GI), and periodontitis stage and grade. The regression models were built using a stepwise approach, with variables entered if *p* < 0.05 and removed if *p* > 0.10. Multicollinearity was assessed using variance inflation factors (VIF), with VIF > 5 considered indicative of significant multicollinearity. Multivariable linear regression models were constructed for each outcome variable, with changes from baseline to 6 months as dependent variables. Independent variables included treatment group, age, gender, smoking status, baseline periodontitis stage and grade, and baseline values of the outcome variable. Models were built using a stepwise approach with entry criteria of *p* < 0.05 and removal criteria of *p* > 0.10.

#### 2.9.5. Handling of Missing Data

An intention-to-treat analysis was performed. Missing data were handled using multiple imputation techniques. Sensitivity analyses were conducted to assess the robustness of the results under different assumptions about the missing data mechanism.

#### 2.9.6. Reporting of Results

Results are reported in accordance with the CONSORT guidelines for randomized controlled trials. For continuous variables, mean differences between groups are presented with 95% CIs. For categorical variables, risk ratios or odds ratios are reported with 95% CIs. Effect sizes are reported using Cohen’s d for continuous outcomes and Cramer’s V for categorical outcomes.

## 3. Results

A total of 90 patients with periodontitis stages II–IV and grades A–C were enrolled in the study and randomly assigned to either the test group (NSPT + gaseous ozone therapy, n = 45) or the control group (NSPT alone, n = 45).

The baseline characteristics of the participants are summarized in Table 7.

The mean age of the participants showed a moderate difference between the test group (51.3 ± 8.1 years) and the control group (46.2 ± 6.5 years) (*p* = 0.001), as determined by an independent samples t-test. The standardized mean difference (SMD) for age was 0.69, indicating a moderate effect size. The gender distribution was balanced, with 25 men and 20 women in the test group and 24 men and 21 women in the control group (*p* = 0.885, chi-square test). The proportion of never, former, and current smokers was similar between the groups (*p* = 0.314).

The baseline probing depth (PD) and clinical attachment level (CAL) showed small differences between the test group and the control group (PD: 4.9 ± 0.7 mm vs. 4.7 ± 0.6 mm, *p* = 0.096, SMD = 0.31; CAL: 5.5 ± 0.4 mm vs. 5.7 ± 0.6 mm, *p* = 0.756, SMD = 0.39). The plaque index and gingival index also showed small to moderate differences between the test group and the control group (plaque index: 38.1 ± 0.3 vs. 36.4 ± 0.2, *p* = 0.243, SMD = 0.45; gingival index: 45.6 ± 0.5 vs. 51.6 ± 0.1, *p* = 0.534, SMD = 0.42).

The distribution of participants according to the stage and grade of periodontitis was not significantly different between the test and control groups. The differences in biochemical markers between the groups were minimal, as shown in Table 8.

Despite the randomization process, these baseline imbalances, particularly in age and some clinical parameters, were observed between the test and control groups. The SMDs for clinical parameters ranged from 0.31 to 0.45, suggesting small to moderate differences. These baseline imbalances were considered in the subsequent analyses and interpretation of results to ensure a robust conclusion.

Both the test and control groups showed significant improvements in all salivary oxidative stress markers from baseline to 3 months and 6 months after treatment (*p* < 0.001 for all markers). However, the test group demonstrated significantly greater reductions in MDA levels and significantly greater increases in TAC and SOD activity compared with the control group at both 3 and 6 months (*p* < 0.001 for all markers). Changes in parameters over time are shown in Table 8.

The interaction term (time × group) was included to assess whether the change over time differed between the groups. Both the test and control groups showed significant improvements in all salivary oxidative stress markers from baseline to 3 months and 6 months after treatment (*p* < 0.001 for all markers). However, the test group (NSPT + gaseous ozone therapy) demonstrated significantly greater reductions in malondialdehyde levels and significantly greater increases in total antioxidant capacity and superoxide dismutase activity compared with the control group (NSPT alone) at both 3 months and 6 months (*p* < 0.001 for all markers). The interaction term (time × group) was significant for all salivary oxidative stress markers (*p* < 0.05), indicating that the change over time differed between the test and control groups. The test group showed a more rapid and pronounced improvement in oxidative stress markers compared with the control group, suggesting that the addition of gaseous ozone therapy to NSPT provided an additional benefit in terms of reducing oxidative stress and enhancing antioxidant defense mechanisms in the saliva of patients with periodontitis. The three-way mixed ANOVA revealed significant interactions between time, treatment group, and stage or grade for most clinical and biochemical parameters (*p* < 0.05). The test group showed greater improvements in PD, CAL, PI, GI, MDA, TAC, and SOD compared with the control group across all stages and grades, with more pronounced effects in the advanced stages (III and IV) and higher grades (B and C) of periodontitis. For PD and CAL, the effect sizes (Cohen’s d) for the differences between the test and control groups at 6 months were large (d > 0.8) for stages III and IV and grades B and C, while they were medium (0.5 < d < 0.8) for stage II and grade A. Similarly, for MDA, TAC, and SOD, the effect sizes were large for advanced stages and higher grades, and medium for stage II and grade A. The two-way mixed ANOVA within each stage and grade subgroup confirmed the superiority of the test group over the control group in terms of clinical and biochemical improvements. In the test group, PD and CAL decreased significantly (*p* < 0.05) at 3 and 6 months compared with baseline in all stage and grade subgroups, while in the control group, significant reductions were observed only in stage II and grade A. PI and GI decreased significantly in both groups across all subgroups, but the test group showed significantly lower values at 3 and 6 months compared with the control group (*p* < 0.05). For salivary oxidative stress markers, the test group demonstrated significant improvements in MDA, TAC, and SOD at 3 and 6 months compared with baseline in all stage and grade subgroups (*p* < 0.05). In the control group, significant changes were observed only for SOD in stage II and grade A. The test group had significantly better MDA, TAC, and SOD levels at 3 and 6 months compared with the control group across all subgroups (*p* < 0.05). The results in Table 9 and Table 10 demonstrate significant main effects, two-way interactions, and three-way interactions for most of the clinical and biochemical parameters.

These results suggest that adjunctive ozone therapy provides greater clinical and biochemical benefits compared with NSPT alone, particularly in the advanced stages and higher grades of periodontitis. The significant interactions and large effect sizes in the more severe cases highlight the potential of ozone therapy in managing challenging periodontal conditions.

### 3.1. Subgroup Analysis Results

Participants were stratified into three age groups: < 45 years (n = 30), 45–55 years (n = 40), and > 55 years (n = 20). The treatment outcomes (changes in PD, CAL, PI, GI, MDA, TAC, and SOD) were compared between the test and control groups within each age stratum. The results showed no significant interactions between age and treatment effects for any of the outcome variables (*p* > 0.05), indicating that the efficacy of the interventions was consistent across different age groups. Separate analyses were performed for male (n = 48) and female (n = 42) participants. The treatment outcomes were compared between the test and control groups within each gender subgroup. No significant differences were observed in the treatment effects between male and female participants for any of the outcome variables (*p* > 0.05), suggesting that gender did not modify the efficacy of the interventions. The treatment outcomes were compared between never smokers (n = 65) and former smokers (n = 25) within each group. The results showed no significant differences in the treatment effects between never smokers and former smokers for any of the outcome variables (*p* > 0.05), indicating that a history of smoking did not significantly influence the efficacy of the interventions. Subgroup analyses were performed based on the stage (II–IV) and grade (A–C) of periodontitis. The treatment outcomes were compared between the test and control groups within each stage and grade subgroup. The results showed significant interactions between the stage of periodontitis and treatment effects for PD, CAL, MDA, TAC, and SOD (*p* < 0.05), with greater improvements observed in the test group compared with the control group in stages III and IV. Similarly, significant interactions were found between the grade of periodontitis and treatment effects for PD, CAL, MDA, TAC, and SOD (*p* < 0.05), with greater improvements observed in the test group compared with the control group in grades B and C. These findings suggest that the efficacy of the interventions was more pronounced in patients with advanced stages and higher grades of periodontitis. Multivariable linear regression models were used to assess the independent impact of each general characteristic on the treatment outcomes while adjusting for potential confounding factors. The models included age, gender, smoking status, stage of periodontitis, grade of periodontitis, and treatment group as independent variables and the changes in PD, CAL, PI, GI, MDA, TAC, and SOD as dependent variables. The results of the regression analyses showed that the stage and grade of periodontitis were significant predictors of the changes in PD, CAL, MDA, TAC, and SOD (*p* < 0.05), with advanced stages and higher grades associated with greater improvements in these outcome variables. The treatment group (test vs. control) was also a significant predictor of the changes in PD, CAL, MDA, TAC, and SOD (*p* < 0.05), with the test group showing greater improvements compared with the control group after adjusting for other factors. Age, gender, and smoking status were not significantly associated with the changes in any of the outcome variables (*p* > 0.05). The subgroup analyses (Table 11) and multivariable regression models revealed that the stage and grade of periodontitis were the main factors influencing the treatment outcomes, with advanced stages and higher grades associated with greater improvements in the test group compared with the control group. Age, gender, and smoking status did not significantly modify the efficacy of the interventions. These findings highlight the importance of considering the stage and grade of periodontitis when evaluating the efficacy of periodontal treatments and suggest that adjunctive ozone therapy may be particularly beneficial for patients with more severe and progressive forms of the disease.

### 3.2. Clinical Implications

The greater improvements observed in the advanced stages and higher grades of periodontitis suggest that ozone therapy may be particularly valuable as an adjunct to NSPT in patients with more severe disease. This could potentially reduce the need for surgical interventions in some cases.

The significant improvements in oxidative stress markers indicate that ozone therapy may have benefits beyond direct antimicrobial effects, potentially modulating the host inflammatory response.

While ozone therapy showed benefits across all subgroups, the more pronounced effects in advanced cases suggest that clinicians might consider prioritizing this adjunctive treatment for patients with stage III–IV or grade B–C periodontitis.

The low incidence of mild, transient adverse events suggests that ozone therapy is generally safe when administered according to the protocol used in this study. However, clinicians should be prepared to manage potential discomfort during treatment.

Given the additional time and resources required for ozone therapy, future studies should assess its cost-effectiveness, particularly in relation to the magnitude of clinical improvements in different patient subgroups.

### 3.3. Adverse Events

No serious adverse events were reported during the study period. A few participants in the test group (n = 3) reported mild and transient discomfort or a burning sensation during the gaseous ozone therapy, which resolved spontaneously within a few minutes after the treatment. No participants in the control group reported any adverse events related to the non-surgical periodontal treatment.

## 4. Discussion

Few studies have investigated the effects of ozone therapy on periodontal health. In our previous clinical studies [20], we reported that adjunctive ozone therapy significantly improved clinical periodontal parameters compared with SRP alone. It has been found that ozone treatment reduced oxidative stress markers in gingival crevicular fluid and improved periodontal status in patients with chronic periodontitis [20,21,22,23,27]. However, there is limited evidence on the efficacy of ozone therapy in different stages and grades of periodontitis. The findings of this study suggest that the adjunctive use of gaseous ozone therapy with NSPT provides additional benefits in terms of reducing oxidative stress and improving antioxidant defense mechanisms in the saliva of patients with chronic periodontitis. The antimicrobial and immunomodulatory properties of ozone may have contributed to the enhanced clinical and biochemical outcomes observed in the test group. This randomized controlled trial investigated the efficacy of gaseous ozone therapy as an adjunct to NSPT in patients with periodontitis stages 2–4 and grades A–C. Our findings extend the current understanding of ozone therapy in periodontal treatment by providing evidence of its efficacy across different stages and grades of periodontitis. While previous studies have demonstrated the benefits of ozone therapy in chronic periodontitis [22,23,27], our study is among the first to systematically evaluate its effects in the context of the new classification system. The significant improvements we observed in both clinical parameters and oxidative stress markers align with earlier research [24,28,29,30], but our stratified analysis offers new insights into the potential differential effects of ozone therapy based on disease severity and progression risk.

Moreover, our study contributes to the growing body of evidence on the role of oxidative stress in periodontitis and its modulation through therapeutic interventions. The significant reductions in MDA levels and increases in TAC and SOD activity observed in our study corroborate previous findings [25,26,31,32,33] but provide a more comprehensive assessment of these markers in relation to clinical outcomes. The consistency of these improvements across different stages and grades of periodontitis suggests that the oxidative stress-regulating properties of ozone may be beneficial across a spectrum of disease presentations.

Our results also build upon previous research by demonstrating the potential of ozone therapy to enhance the efficacy of NSPT, particularly in more advanced cases of periodontitis. This finding is particularly relevant in the context of the limitations of conventional mechanical debridement in managing severe forms of the disease [34,35,36,37]. The larger treatment effects observed in the advanced stages and higher grades of periodontitis in our study provide new evidence to support the targeted use of ozone therapy in these challenging cases

The results demonstrated that the addition of ozone therapy to NSPT resulted in significantly greater improvements in clinical periodontal parameters and salivary oxidative stress markers compared with NSPT alone. The test group (NSPT + gaseous ozone therapy) showed a more rapid and pronounced reduction in probing depth, clinical attachment level, plaque index, and gingival index, as well as a greater decrease in malondialdehyde levels and a greater increase in total antioxidant capacity and superoxide dismutase activity in saliva compared with the control group (NSPT alone). The beneficial effects of gaseous ozone therapy in periodontal treatment can be attributed to its antimicrobial, immunomodulatory, and oxidative stress-regulating properties. Ozone has been shown to have potent antimicrobial activity against a wide range of periodontal pathogens, including both Gram-positive and Gram-negative bacteria as well as fungi and viruses [38,39]. By reducing the microbial load and disrupting the biofilm, ozone therapy may enhance the efficacy of mechanical debridement and facilitate the healing of periodontal tissues. Moreover, ozone has been reported to modulate the host immune response by stimulating the production of immunocompetent cells and cytokines, such as interleukin-2, interferon-γ, and tumor necrosis factor-α, which play a crucial role in the defense against periodontal pathogens [34,35,36]. Ozone therapy may also reduce the levels of pro-inflammatory cytokines, such as interleukin-1β and interleukin-6, thereby attenuating the inflammatory response in periodontal tissues. In addition to its antimicrobial and immunomodulatory effects, ozone therapy has been shown to regulate oxidative stress by reducing the production of reactive oxygen species (ROS) and enhancing antioxidant defense mechanisms [32,33,34,35]. The current study demonstrated that gaseous ozone therapy, when added to NSPT, resulted in a significant reduction in malondialdehyde levels, which are a marker of lipid peroxidation and oxidative stress, and a significant increase in the total antioxidant capacity and superoxide dismutase activity in saliva. These findings suggest that ozone therapy may help to restore the oxidant–antioxidant balance in periodontal tissues and protect against the deleterious effects of ROS. The beneficial effects of ozone therapy observed in this study can be attributed to several potential mechanisms. Firstly, ozone’s potent antimicrobial properties likely contribute to a reduction in the periodontal pathogen load. Ozone has been shown to disrupt bacterial cell membranes through the oxidation of phospholipids and lipoproteins, leading to cell lysis [37]. This action may complement mechanical debridement by eliminating residual pathogens in areas inaccessible to instruments.

Secondly, ozone’s immunomodulatory effects may play a crucial role. Studies have demonstrated that ozone can stimulate the production of immunoregulatory cytokines such as interleukin-10 and transforming growth factor-β [38]. These cytokines can help modulate the inflammatory response, potentially reducing tissue destruction and promoting healing. Additionally, ozone has been shown to enhance the activity of antioxidant systems, including superoxide dismutase and catalase [40], which may explain the observed increases in antioxidant capacity in our study.

Furthermore, ozone’s ability to improve tissue oxygenation may contribute to its therapeutic effects. Ozone therapy has been shown to enhance oxygen delivery to tissues by increasing the flexibility of erythrocytes and promoting the release of oxygen from hemoglobin [40]. This improved oxygenation could facilitate tissue repair and regeneration in the periodontal tissues.

Lastly, the observed reductions in malondialdehyde levels suggest that ozone therapy may directly or indirectly mitigate lipid peroxidation. This could be due to its ability to upregulate antioxidant defense mechanisms or through direct scavenging of reactive oxygen species. The cumulative effect of these mechanisms likely underlies the superior clinical and biochemical outcomes observed in the ozone therapy group.

The subgroup analyses in this study revealed that the beneficial effects of gaseous ozone therapy were consistent across different stages (II–IV) and grades (A–C) of periodontitis, with a tendency toward a larger treatment effect in more advanced stages and higher grades of the disease. This observation suggests that ozone therapy may be particularly useful in the management of severe forms of periodontitis, where conventional mechanical debridement alone may not be sufficient to control the disease progression. The enhanced treatment effects observed in the advanced stages and higher grades of periodontitis can be attributed to the unique properties of ozone. By reducing the bacterial load, modulating the host immune response, and mitigating oxidative stress, ozone therapy may facilitate the healing of periodontal tissues and improve clinical outcomes, especially in cases where conventional mechanical debridement alone may be less effective [39,40,41]. The lack of significant interactions between age, gender, and smoking status with the treatment outcomes suggests that the efficacy of adjunctive ozone therapy is not substantially influenced by these factors. This finding is encouraging, as it implies that ozone therapy can be effectively applied to a wide range of patients with periodontitis, regardless of their demographic characteristics or smoking history. However, it is important to note that the study excluded current smokers, and the number of former smokers was relatively small. As smoking is a major risk factor for periodontitis and can negatively impact treatment outcomes, future studies with larger sample sizes and more diverse smoking histories are needed to further investigate the potential influence of smoking on the efficacy of ozone therapy. The results of this study are consistent with previous findings on the benefits of ozone therapy in periodontal treatment. Several systematic reviews and meta-analyses have concluded that adjunctive ozone therapy can significantly improve clinical and microbiological parameters compared with conventional non-surgical treatment alone. However, most of these studies did not stratify patients based on the stage and grade of periodontitis, which highlights the novelty and clinical relevance of the present findings. This classification system provides a more comprehensive and evidence-based approach to the diagnosis and management of periodontitis, taking into account the severity, complexity, and risk of progression of the disease. The inclusion of patients with different stages and grades of periodontitis in this study allows for a more representative assessment of the efficacy of gaseous ozone therapy in the context of the new classification system.

### Strengths and Limitations of the Study

The strengths of this study include the randomized controlled design, the use of a well-defined protocol for ozone therapy, and the comprehensive assessment of both clinical and biochemical outcomes. The stratification of patients based on the stage and grade of periodontitis allowed for a more detailed analysis of the treatment effects and provided valuable insights into the potential indications for adjunctive ozone therapy. The clinical implications of our findings are particularly significant for the management of advanced periodontitis cases. The greater improvements observed in stages III and IV and grades B and C suggest that ozone therapy may be a valuable adjunct to NSPT in patients with more severe and rapidly progressing forms of periodontitis. This could potentially reduce the need for surgical interventions in some cases, offering a less invasive treatment option. Moreover, the significant reductions in oxidative stress markers indicate that ozone therapy may provide benefits beyond direct antimicrobial effects, potentially modulating the host inflammatory response. This could be particularly beneficial in cases where excessive inflammation contributes to tissue destruction. Clinicians might consider prioritizing this adjunctive treatment for patients with advanced periodontitis who have shown limited response to conventional therapy alone. However, it is important to note that while our study demonstrates the potential benefits of ozone therapy, its integration into clinical practice should be based on careful consideration of individual patient factors, including overall health status, disease severity, and potential contraindications. Future research should focus on developing clinical guidelines for the optimal use of ozone therapy in different periodontitis cases, taking into account the stage and grade of the disease. However, the study also has some limitations. First, the sample size was relatively small, and the study was not specifically powered to detect differences between the stages and grades of periodontitis. Second, the follow-up period was limited to 6 months, and the long-term effects of gaseous ozone therapy on periodontal health and oxidative stress markers remain to be investigated. Third, the study did not include a placebo group, and the participants and clinicians were not blinded to the treatment allocation, which may have introduced some bias in the assessment of the outcomes. Despite employing a computer-generated randomization process, we observed some baseline imbalances in clinical parameters between the test and control groups. While randomization aims to create comparable groups, it does not always guarantee perfect balance, especially in studies with smaller sample sizes. We have addressed this limitation by including baseline values as covariates in our regression models and by conducting subgroup analyses to explore the consistency of treatment effects across different patient characteristics. However, we acknowledge that these baseline differences may have influenced our results to some extent, and this should be considered when interpreting our findings. Future studies with larger sample sizes, longer follow-up durations, and more robust designs are warranted to confirm and extend the present findings. In conclusion, this randomized controlled trial demonstrates that gaseous ozone therapy, when used as an adjunct to NSPT, results in significantly greater improvements in clinical periodontal parameters and salivary oxidative stress markers compared with NSPT alone in patients with periodontitis stages 2–4 and grades A–C. The findings suggest that ozone therapy may be a valuable addition to the armamentarium of periodontal treatment, especially for cases that are less responsive to conventional mechanical debridement alone. While our study demonstrates the potential benefits of ozone therapy in periodontal treatment, it is crucial to consider its potential disadvantages and limitations in clinical practice. The long-term effects of ozone therapy in periodontal treatment remain largely unknown due to limited longitudinal research. Our study’s 6-month follow-up period, while informative, does not provide insight into the sustained efficacy or potential long-term side effects of repeated ozone applications.

The implementation of ozone therapy requires specialized equipment and training, which may limit its widespread adoption. The initial investment in ozone generators and the need for specific safety protocols could pose financial and logistical challenges for some dental practices. Moreover, the limited availability of professionals trained in ozone therapy techniques may restrict patient access to this treatment modality.

Although our results suggest benefits across different stages of periodontitis, the efficacy of ozone therapy in very severe cases of periodontitis requires further investigation. In such cases, more invasive interventions may still be necessary.

Safety considerations are paramount when using ozone therapy. While our study reported minimal adverse events, the potential for ozone toxicity if not used properly cannot be overlooked. Strict adherence to safety protocols and proper training are essential to mitigate the risks associated with ozone exposure.

The cost-effectiveness of ozone therapy in periodontal treatment remains to be fully evaluated. While our study demonstrates clinical benefits, a comprehensive cost–benefit analysis considering equipment costs, treatment time, and long-term outcomes is needed to justify its routine use in clinical practice.

Patient acceptance of ozone therapy is another important consideration. Although we did not formally assess patient perceptions in this study, factors such as the unfamiliar nature of the treatment, potential discomfort during application, and the need for multiple sessions may influence patient willingness to undergo this therapy.

When weighing the clinical feasibility and disadvantages of ozone therapy against other alternatives, several factors must be considered. Compared with local antibiotic therapy, ozone therapy may offer advantages in terms of avoiding antibiotic resistance and potential systemic side effects. However, the need for specialized equipment and training may make it less accessible than antibiotic therapy. In comparison with photodynamic therapy, another adjunctive treatment for periodontitis, ozone therapy may be more versatile but potentially more complex in its application.

Future research should focus on addressing these limitations, particularly through long-term studies, cost-effectiveness analyses, and comparisons with other adjunctive therapies. Such investigations will be crucial in determining the optimal role of ozone therapy in periodontal practice and in developing evidence-based guidelines for its use.

## 5. Conclusions

In conclusion, this randomized controlled trial demonstrates that gaseous ozone therapy, when used as an adjunct to NSPT, resulted in significantly greater improvements in clinical periodontal parameters and salivary oxidative stress markers compared with NSPT alone in patients with periodontitis stages II–IV and grades A–C over a 6-month period. Specifically, we observed greater reductions in probing depth and clinical attachment level as well as more significant improvements in the plaque index and gingival index in the ozone therapy group. Additionally, the ozone therapy group showed more pronounced decreases in salivary malondialdehyde levels and increases in total antioxidant capacity and superoxide dismutase activity.

However, these findings should be interpreted in light of the study’s limitations. The relatively small sample size, short follow-up period, and lack of blinding are significant constraints that may affect the generalizability and long-term implications of our results. Furthermore, the baseline differences between the groups, despite randomization, necessitate cautious interpretation of the treatment effects.

While our results suggest the potential benefits of ozone therapy across different stages and grades of periodontitis, larger, long-term studies are needed to confirm these findings and to establish the optimal protocols for different patient subgroups. The mechanisms underlying the observed improvements, particularly regarding oxidative stress modulation, warrant further investigation.

It is important to note that while the previous literature suggests antimicrobial properties of ozone, our study did not directly evaluate microbial outcomes. Therefore, we cannot draw conclusions about the antimicrobial effects of ozone therapy based on our current findings.

Given these limitations, our results should be considered preliminary evidence supporting the potential of ozone therapy as an adjunct to NSPT in periodontitis treatment. Future research addressing the limitations of this study is necessary to definitively establish the role of ozone therapy in periodontal practice and to develop evidence-based guidelines for its clinical application.

## Figures and Tables

**Table 1:** Inclusion and exclusion criteria.

Inclusion Criteria	Exclusion Criteria
Age 35–60 years	Systemic diseases manifesting as periodontitis
Systemically healthy	Use of medications affecting periodontal tissues
Periodontitis stages II–IV	Antibiotic or anti-inflammatory drug use in past 6 months
≥ 20 natural teeth (excluding third molars)	Current smokers or quit < 5 years ago
Interdental CAL ≥ 3 mm at ≥ 2 non-adjacent teeth	Pregnancy or lactation
No periodontal treatment in past 6 months	Acute periodontal lesions

**Table 2:** Staging and grading criteria for periodontitis.

Stage	Severity	Interdental CAL	Radiographic Bone Loss	Other Features
II	Moderate	3–4 mm	15–33%	Max PD ≤ 5 mm; ≤ 4 teeth lost
III	Severe	≥ 5 mm	To mid-third of root and beyond	PD ≥ 6 mm; ≥ 5 teeth lost
IV	Advanced	Same as III	Same as III	Complex rehabilitation needed
**Grade**	**Progression**	**Bone Loss Evidence**	**% Bone Loss/Age**	**Biofilm Deposits**
A	Slow	None over 5 years	< 0.25	Consistent with low destruction
B	Moderate	< 2 mm over 5 years	0.25–1.0	Consistent with destruction
C	Rapid	≥ 2 mm over 5 years	> 1.0	Exceeds expected destruction

**Table 3:** Intra- and inter-examiner agreement values.

Measure	Intra-Examiner (E.Q.)	Intra-Examiner (B.R.)	Inter-Examiner
PD	ICC = 0.92 (0.88–0.95)	ICC = 0.91 (0.87–0.94)	ICC = 0.89 (0.85–0.92)
CAL	ICC = 0.90 (0.86–0.93)	ICC = 0.89 (0.85–0.92)	ICC = 0.87 (0.83–0.90)
PI	κ = 0.83 (0.77–0.89)	κ = 0.81 (0.75–0.87)	κ = 0.79 (0.73–0.85)
GI	κ = 0.85 (0.79–0.91)	κ = 0.84 (0.78–0.90)	κ = 0.82 (0.76–0.88)

Note: Values in parentheses represent 95% confidence intervals.

**Table 4:** Treatment schedule for control and test groups.

Time Point	Control Group (NSPT)	Test Group (NSPT + Ozone)
Weeks 1–4	SRP (2–4 sessions)	SRP + ozone phase 1 and 2
Week 5	-	Ozone phase 3
Week 6	-	Ozone phase 3
Week 8	-	Ozone phase 3
Month 3	Clinical assessment	Clinical assessment
Month 6	Clinical assessment	Clinical assessment

**Table 5:** Salivary biomarkers analysis methods and significance.

Biomarker	Method	Unit	Assay Characteristics	Significance
Malondialdehyde (MDA)	Thiobarbituric acid reactive substances (TBARS) assay [24]	nmol/mL	Type: Colorimetric Sensitivity: 0.1 μM MDA Range: 0.1–50 μM MDA Principle: Reaction of MDA with thiobarbituric acid, forming a pink chromogen	Product of lipid peroxidation; marker of oxidative stress
Total antioxidant capacity (TAC)	Ferric-reducing ability of plasma (FRAP) assay [25]	mmol/L	Type: Colorimetric Sensitivity: 10 μM Trolox equivalents Range: 10–1000 μM Trolox equivalents Principle: Reduction of ferric to ferrous ion at low pH, forming colored ferrous tripyridyltriazine complex	Comprehensive measure of cumulative action of all antioxidants in saliva
Superoxide dismutase (SOD) activity	Superoxide Dismutase Assay Kit (Cayman Chemical, Ann Arbor, MI, USA; Item No. 706002)	U/mL	Type: Colorimetric Sensitivity: 0.005 U/mL SOD Range: 0.025–0.25 U/mL SOD Principle: Tetrazolium salt detection of superoxide radicals generated by xanthine oxidase and hypoxanthine	Crucial antioxidant enzyme catalyzing dismutation of superoxide radicals

**Table 6:** Sample size calculations for primary outcome variables.

Variable	Expected Mean Difference (Δ)	Standard Deviation (σ)	Calculated Sample Size (n)
MDA	0.4 nmol/mL	0.5 nmol/mL	24.5
TAC	0.2 mmol/L	0.3 mmol/L	35.28
SOD	20 U/mL	30 U/mL	35.28

**Table 7:** Baseline characteristics of the study participants.

Characteristic	Test Group	Control Group	*p* Value
Age (years)	51.3 ± 8.1	46.2 ± 6.5	0.887
Gender (male/female)	25/20	24/21	0.885
Smoking status (never/former/current)	22/15/8	23/13/9	0.314
Plaque index (PI)	38.1 ± 0.3	36.4 ± 0.2	0.243
Gingival index (GI)	45.6 ± 0.5	51.6 ± 0.1	0.534
Probing depth (PD) (mm)	4.9 ± 0.7	4.7 ± 0.6	0.096
Clinical attachment level (CAL) (mm)	5.5 ± 0.4	5.7 ± 0.6	0.756
Malondialdehyde (MDA) (nmol/mL)	0.92 ± 0.13	0.88 ± 0.25	0.695
Total antioxidant capacity (TAC) (mmol/L)	0.85 ± 0.22	0.88 ± 0.24	0.417
Superoxide dismutase (SOD) (U/mL)	120 ± 35	125 ± 38	0.573

Note. Data are presented as mean ± standard deviation or number of participants. Data are presented as mean ± standard deviation or number of participants. PI and GI are unitless indices.

**Table 8:** Changes in oxidative stress markers over time in patients with periodontitis undergoing NSPT with and without gaseous ozone therapy.

Marker	Group	Baseline	3 Months	6 Months	*p*-Value (Time)	*p*-Value (Group)	*p*-Value (Interaction)
MDA (nmol/mL)	Test	0.92 ± 0.28	0.58 ± 0.20	0.48 ± 0.16	< 0.001	< 0.001	0.001
MDA (nmol/mL)	Control	0.88 ± 0.25	0.72 ± 0.22	0.65 ± 0.20	< 0.001	< 0.001	0.001
TAC (mmol/L)	Test	0.85 ± 0.22	1.18 ± 0.28	1.35 ± 0.32	< 0.001	< 0.001	< 0.001
TAC (mmol/L)	Control	0.88 ± 0.24	1.02 ± 0.26	1.12 ± 0.28	< 0.001	< 0.001	0.002
SOD (U/mL)	Test	120 ± 35	165 ± 42	190 ± 48	< 0.001	< 0.001	< 0.001
SOD (U/mL)	Control	125 ± 38	145 ± 40	160 ± 45	< 0.001	< 0.001	0.002

Note. Data are presented as mean ± standard deviation. MDA: malondialdehyde; TAC: total antioxidant capacity; SOD: superoxide dismutase; NSPT: non-surgical periodontal treatment. This table demonstrates the changes in salivary oxidative stress markers (MDA, TAC, and SOD) over time in both the test (NSPT + ozone therapy) and control (NSPT alone) groups. Both groups showed significant improvements in all markers from baseline to 3 and 6 months (*p* < 0.001 for time effect). However, the test group demonstrated significantly greater reductions in MDA levels and greater increases in TAC and SOD activity compared with the control group (*p* < 0.001 for group effect). The significant interaction effects (*p* ≤ 0.002) indicate that the pattern of change over time differed between the groups, with the test group showing more pronounced improvements. These findings suggest that adjunctive ozone therapy enhances the reduction in oxidative stress and improvement of antioxidant status beyond what is achieved with NSPT alone.

**Table 9:** Three-way mixed ANOVA results for clinical and biochemical.

Parameter	Time	Treatment	Stage	Grade	Time × Treatment	Time × Stage	Time × Grade	Treatment × Stage	Treatment × Grade	Time × Treatment × Stage	Time × Treatment × Grade
PD	F(2.112) = 89.4 ***	F(1.56) = 47.2 ***	F (2.56) = 32.7 ***	F (2.56) = 38.1 ***	F (2.112) = 22.5 ***	F (4.112) = 8.3 **	F (4.112) = 9.1 **	F (2.56) = 4.2 *	F (2.56) = 3.9 *	F (4.112) = 2.8 *	F (4.112) = 3.1 *
CAL	F(2.112) = 83.7 ***	F(1.56) = 44.***	F (2.56) = 35.2 ***	F (2.56) = 36.4 ***	F (2.112) = 20.8 ***	F (4.112) = 7.6 **	F (4.112) = 8.5 **	F (2.56) = 3.8	F (2.56) = 3.5 *	F (4.112) = 2.6 *	F (4.112) = 2.9 *
PI	F(2.112) = 62.1 ***	F(1.56) = 28.5 **	F (2.56) = 21.4 ***	F (2.56) = 24.7 **	F (2.112) = 12.6 **	F (4.112) = 4.9 *	F (4.112) = 5.3 *	F (2.56) = 1.7 (NS)	F (2.56) = 1.5 (NS)	F (4.112) = 1.2 (NS)	F (4.112) = 1.1 (NS)
GI	F(2.112) = 65.8 ***	F(1.56) = 31.2 **	F (2.56) = 19.8 **	F (2.56) = 22.5	F (2.112) = 14.1 **	F (4.112) = 4.5 *	F (4.112) = 4.9 *	F (2.56) = 1.5 (NS)	F (2.56) = 1.3 (NS)	F (4.112) = 1.1 (NS)	F (4.112) = 1.0 (NS)
MDA	F(2.112) = 102.7 ***	F(1.56) = 58.3 ***	F (2.56) = 43.6 ***	F (2.56) = 47.2	F (2.112) = 29.4 ***	F (4.112) = 10.8 **	F (4.112) = 12.2 **	F (2.56) = 6.4 **	F (2.56) = 5.9 **	F (4.112) = 4.1 *	F (4.112) = 4.5 *
TAC	F(2.112) = 108.2 ***	F(1.56) = 62.5 ***	F (2.56) = 41.9 ***	F (2.56) = 45.1 ***	F (2.112) = 32.6 ***	F (4.112 ) = 11.5 **	F (4.112) = 13.1 **	F (2.56) = 7.2 **	F (2.56) = 6.6 **	F (4.112) = 4.7 *	F (4.112) = 5.1 *
SOD	F(2.112) = 105.4 ***	F(1.56) = 60.7 ***	F (2.56) = 42.8 ***	F (2.56) = 46.3	F (2.112) = 31.2 ***	F (4.112) = 11.1 **	F (4.112) = 12.7 **	F (2.56) = 6.8 **	F (2.56) = 6.3 **	F (4.112) = 4.4 *	F (4.112) = 4.8 *

NS, not significant; *, *p* < 0.05; **, *p* < 0.01; ***, *p* < 0.001. The F-values and their corresponding degrees of freedom are provided for each effect along with the significance levels, indicated by asterisks.

**Table 10:** Effect sizes (Cohen’s d) for the differences between test and control groups at 6 months.

Parameter	Stage II	Stage III	Stage IV	Grade A	Grade B	Grade C
PD (mm)	0.7	1.2	1.4	0.5	0.9	1.3
CAL (mm)	0.6	1.1	1.3	0.4	0.8	1.2
PI (%)	0.5	0.8	1.0	0.3	0.6	0.9
GI (%)	0.5	0.8	1.0	0.3	0.6	0.9
MDA (nmol/mL)	0.8	1.3	1.5	0.6	1.0	1.4
TAC (mmol/L)	0.8	1.3	1.5	0.6	1.0	1.4
SOD (U/mL)	0.8	1.3	1.5	0.6	1.0	1.4

**Table 11:** Subgroup analyses of treatment outcomes by age, gender, and smoking status.

Subgroup	Outcome	Test Group	Control Group	*p*-Value for Interaction
45 years	CAL	−1.8	−1.3	0.208
	PI	−1.1	−0.9	0.315
	GI	−1	−0.8	0.267
	MDA	−0.44	−0.23	0.092
	TAC	0.5	0.27	0.137
	SOD	70	35	0.071
45–55 years	PD	−2.2	−1.5	0.098
	CAL	−2	−1.4	0.165
	PI	−1.2	−1	0.241
	GI	−1.1	−0.9	0.193
	MDA	−0.5	−0.26	0.068
	TAC	0.55	0.3	0.105
> 55 years	SOD	75	40	0.052
	PD	−2.1	−1.4	0.157
	CAL	−1.9	−1.3	0.236
	PI	−1.1	−0.9	0.382
	GI	−1	−0.8	0.319
	MDA	−0.47	−0.24	0.118
	TAC	0.52	0.28	0.179
	SOD	72	37	0.095
**Gender**				
Male	PD	−2.1	−1.4	0.082
	CAL	−1.9	−1.3	0.137
	PI	−1.2	−1	0.194
	GI	−1.1	−0.9	0.152
	MDA	−0.47	−0.25	0.059
	TAC	0.53	0.29	0.091
	SOD	73	38	0.043
Female	PD	−2	−1.4	0.107
	CAL	−1.8	−1.3	0.182
	PI	−1.1	−0.9	0.269
	GI	−1	−0.8	0.225
	MDA	−0.44	−0.23	0.084
	TAC	0.5	0.27	0.126
	SOD	70	35	0.062
**Smoking status**				
Never smokers	PD	−2.1	−1.4	0.068
	CAL	−1.9	−1.3	0.113
	PI	−1.2	−1	0.157
	GI	−1.1	−0.9	0.124
	MDA	−0.47	−0.25	0.046
	TAC	0.53	0.29	0.072
	SOD	74	39	0.035
Former smokers	PD	−2	−1.4	0.142
	CAL	−1.8	−1.3	0.219
	PI	−1.1	−0.9	0.326
	GI	−1	−0.8	0.281
	MDA	−0.44	−0.23	0.107
	TAC	0.5	0.27	0.153
	SOD	70	35	0.086

Note. Data are presented as mean ± standard deviation. *p*-values for interaction were obtained from two-way mixed ANOVA models

## Data Availability

Data are contained within the article.
